# Single Nucleotide Polymorphisms in Oxidative Stress-Related Genes Are Associated with Autism Spectrum Disorders

**DOI:** 10.3390/ijms26199768

**Published:** 2025-10-07

**Authors:** Giulia Spoto, Maria Paola Bertuccio, Giuseppa Visalli, Monica Currò, Gabriella Di Rosa, Daniela Caccamo

**Affiliations:** Department of Biomedical Sciences, Dental Sciences, and Morpho-Functional Imaging, University Hospital “G. Martino”, 98124 Messina, Italy; giulia.spoto27@gmail.com (G.S.); mariapaola.bertuccio@unime.it (M.P.B.); giuseppa.visalli@unime.it (G.V.); monica.curro@unime.it (M.C.); gdirosa@unime.it (G.D.R.)

**Keywords:** autism spectrum disorders, oxidative stress markers, single nucleotide polymorphisms, genotyping

## Abstract

Autism spectrum disorder (ASD) is a complex group of severe neurodevelopmental disorders characterized by varying degrees of dysfunctional communication and social abilities as well as repetitive and compulsive stereotypic behaviors. We aim to evaluate the genetic predisposition to oxidative response and its relationship with altered oxidative stress markers in ASD patients. Genomic DNA was isolated from peripheral blood lymphocytes of 106 (83 M, 23 F; 7.9 ± 3.2 years) ASD patients and 90 healthy subjects (63 M, 27 F; 21.2 ± 1.8 years). Genotyping was performed by real-time PCR-based allelic discrimination, PCR and electrophoresis of GST deletion variants. Reactive oxygen metabolites (dROMs), the Biological Antioxidant Potential (BAP), and the advanced oxidation protein products (AOPP) were also measured. Furthermore, we assessed oxidative DNA damage by Single Cell Gel Electrophoresis. The evaluation of oxidative stress markers indicated a mild oxidative stress status and a higher level of DNA damage in nuclei of ASD patients’ lymphocytes. We found significant associations between ASD and several polymorphisms of genes involved in the detoxification and the response to oxidative stress. Genetic and environmental factors contribute to the onset of autism spectrum disorder, and ASD patients’ treatment requires a multimodal approach, including behavioral, educational, and pharmacological approaches.

## 1. Introduction

Autism spectrum disorder (ASD) is a complex group of severe neurodevelopmental disorders, characterized by persistent impairment in communication and social abilities, including social-emotional reciprocity and nonverbal communicative behaviors, as well as repetitive and restrictive patterns of behaviors and interests, such as stereotyped movements and speech and abnormalities in sensory processing [1]. A current estimation of ASD prevalence shows that 1 in 44 children is affected by ASD, and boys are three–four times more affected than girls [2,3].

Although ASD etiology and pathophysiology have not been well understood, neurobiological studies in ASD animal models, as well as neurophysiological, anatomical, and non-invasive brain imaging studies, have highlighted alterations of pathways involved in neural development, synapse plasticity, structural brain abnormalities, cognition, and behavior [3]. Several twin studies demonstrated the heritability of autism having this an estimated range from 64% to 93% [4,5,6]. Moreover, genome studies have demonstrated the presence and heritability of a variety of genetic abnormalities, mostly represented by mutations in genes encoding for proteins involved in neuronal and synaptic pathways, in patients affected by ASD [7]. Although ASD can, in some cases, be attributed to monogenic disorders, such as those related to pathogenic variants in SHANK3, MECP2, FOXG1, CDKL5, PTEN, and FMR1, the majority of cases are thought to result from the combined effects of multiple common and rare genetic variants, interacting with environmental factors [8,9,10]. Due to sample heterogeneity and differences in analysis method used, heritability of common genetic variants such as single nucleotide polymorphisms (SNPs) showed a considerable variability, ranging from 65% in multiplex families [11] to 12% in the Psychiatric Genomics Consortium GWAS (genome-wide association studies) [12]. Moreover, accumulating evidence showed that the penetrance of risk genes is modulated by epigenetic factors, such as DNA methylation, non-coding RNAs, and histone modifications, and that environmental factors (i.e., maternal diet and exposure to drugs or environmental toxicants) also play a key role in increasing the susceptibility to ASD, above all because of their ability to determine epigenetic changes [13,14,15,16,17,18].

Interestingly, in recent decades, research and clinical studies focused on ASD-related specific organ dysfunctions, such as immune dysregulation, inflammation, impaired detoxification, environmental toxicant exposures, redox regulation/oxidative stress, and energy generation/mitochondrial systems [19,20]. Oxidative stress and inflammation play significant roles in contributing to mutagenesis, particularly in vulnerable genomic regions associated with conditions like ASD [21]. These processes heighten susceptibility to DNA damage and mutations, which can have profound implications for genomic stability and the development of neurological disorders, especially at early age of brain development [22,23]. Moreover, a role for maternal immune system dysregulation, including alterations of cytokine/chemokine secretion and the presence of maternal autoantibodies against proteins active in the developing brain, has been recognized as a prominent risk factor for ASD development in the offspring [24,25,26].

As a result of the cumulative influence of toxic environmental insults, oxidative stress may develop and play a central role in ASD pathogenesis by promoting neuronal damage in several ways, such as lipid peroxidation, protein carbonylation, and DNA oxidation [27]. Post-mortem analysis of brain tissues from autistic cases showed higher levels of oxidative stress biomarkers than in healthy controls [28,29]. Moreover, mitochondrial abnormalities—a potential source of elevated oxidative stress—were reported in autistic case studies and have been recently re-evaluated through meta-analysis [30,31].

Several case–control studies have reported lower concentrations of reduced glutathione (GSH), higher levels of oxidized glutathione (GSSG), and a decrease in the GSH/GSSG redox ratio, along with a lower mitochondrial GSH reserve in ASD patients compared with healthy subjects. In addition, in some studies lower GSH levels and markers of increased oxidative stress have been correlated with ASD severity [32]. Markers of oxidative stress have also been correlated with the severity of gastrointestinal problems in individuals with ASD [19].

Here, we aimed to assess the distribution of genetic polymorphisms in gene encoding for phase I and phase II detoxification enzymes, which have the task of metabolizing drugs and xenobiotics, as well as antioxidant enzymes in ASD patients and healthy individuals. Phase I enzymes, such as CYP450 enzymes, usually catalyze reactions of oxidation, re-duction and hydrolysis, while phase II enzymes, such as glutathione S-transferases (GSTs), UDP-glucuronosyltransferases (UGTs) and sulfotransferases (SULTs) catalyze conjugation reactions [33]. Moreover, the relationships between individual genetic background and alterations of oxidative stress markers were also examined in ASD patients and case–controls.

## 2. Results

### 2.1. Genotyping for SNPs in Antioxidant Defense Enzymes

Individuals with ASD and controls were genotyped for the SNPs Superoxide Dismutase 2 (SOD2) A16V, Catalase (CAT) -844 C>T, Glutathione peroxidase 1 (GPx1) rs1800668, and Paraoxonase 1 (PON1) Q192R.

Genotype frequencies for the SNP SOD2 A16V were found to be in Hardy–Weinberg equilibrium both in patients and controls. The most frequent genotype in ASD group was the heterozygous AV, while the mutated variant V was more frequent in controls than in ASD group.

Genotype frequencies for the SNP CAT -844 C>T were in Hardy–Weinberg equilibrium in individuals with ASD and controls. Wild-type genotype was more common in controls compared to ASD, with a prevalence around 70%. Heterozygous genotype was statistically significant higher in patients than in controls. Neither affected individuals nor the controls resulted carriers of the homozygous mutated genotype TT.

Results from allelic discrimination for the SNP GPx1 rs1800668 showed that the frequencies were in Hardy–Weinberg equilibrium, both in patients and controls.

The frequency of wild-type genotype was significantly higher in controls, while the homozygous mutated genotype was observed only in individuals with ASD. Notably, Odds Ratio (OR) calculation showed that the mutated genotype was associated with a 23 times higher risk to develop ASD (Table 1).

The distributions of genotypes for the SNP PON1 Q192R in ASD patients and controls were in Hardy–Weinberg equilibrium.

The frequency of wild-type genotype was significantly higher in healthy controls than in cases, while the homozygous mutated genotype was only observed in individuals with ASD, and was associated with a 31 times higher risk for the disease. On the contrary, the QQ wild-type genotype resulted in being protective against ASD and associated with a two times lower risk for the disease (Table 1).

### 2.2. Genotyping of Polymorphisms in Enzymes and Proteins Involved in Xenobiotic Metabolism

ASD patients and healthy individuals were genotyped for the cytochrome P450 (CYP), family 2, subfamily C, polypeptide 9 and 19, and subfamily D, polypeptide 6, namely SNPs CYP2C9*2, CYP2C9*3, CYP2C19*2, CYP2D6*41 of phase I drug metabolism enzymes CYP2C9, CYP2C19, CYP2D6, the SNP Arg554Lys of the xenobiotic sensor aryl hydrocarbon receptor (AHR), and the SNPs Arg187Gln, rs179993, * 6, I104V as well as A114V of the phase II drug metabolism enzymes N-acetyl transferases (NATs), UDP-glucuronosyl transferases (UGTs), glutathione-S-transferases (GSTs): NAT1, NAT2, UGT1A1, GSTP1, respectively. Moreover, the distribution of deletion variants of the phase II drug metabolism enzymes GSTM1 and GSTT1 was also assessed in both groups.

Genotype distributions for CYP2C9*2 and *3 polymorphisms were in Hardy–Weinberg equilibrium in both patients and controls.

The CYP2C9*1/*1 wild-type genotype was the most frequent in both groups, with a higher but not significant frequency in control group. The mutated *1/*2 was more frequent among healthy controls than among cases, even if these differences were not statistically significant. The homozygous mutated *2/*2 genotype showed a very low frequency in both groups. Notably, the mutated heterozygous *1/*3 genotype was significantly more frequent in ASD patients than in healthy subjects and was associated with a 20 times increased risk for the disease (Table 2). The mutated homozygous *3/*3 genotype was not observed in both groups.

Genotype distribution for the CYP2C19 *2 polymorphism agreed with Hardy–Weinberg equilibrium both in patients and controls.

The wild-type *1/*1 genotype was more frequent among healthy subjects than among ASD patients, while the mutated heterozygous *1/*2 and homozygous *2/*2 genotypes were more frequent in cases than in controls. However, these differences were not statistically significant (Table 2).

Genotype distribution for the CYP2D6*41 polymorphism was in agreement with Hardy–Weinberg equilibrium.

The wild-type *1/*1 genotype was the most frequent in both groups, with a significantly higher frequency among controls, that resulted in a reduction in the risk for the disease by 53 times. The *41 mutated allele was only found in ASD group, with the highest frequency of the heterozygous *1/*41 genotype, that was associated with a 44 times increased risk for the disease.

Genotyping for AHR Arg554Lys polymorphism showed that the genotype frequencies were in Hardy–Weinberg equilibrium.

The wild-type Arg/Arg genotype was the most frequent in both groups, with a higher frequency in ASD patients. However, this difference was not statistically significant. The heterozygous mutated Arg/Lys genotype showed a similar frequency between the two groups, while the homozygous mutated Lys/Lys genotype was only found in control group.

The genotype distributions for the SNP NAT1 Arg187Gln and NAT2 was in agreement with Hardy–Weinberg equilibrium both in patients and controls. Genotype distributions were similar between the two groups, with the wild-type Arg/Arg genotype being the most represented and the heterozygous Arg/Gln genotype showing a very low frequency. The mutated homozygous genotype was not observed (Table 2).

The distribution of the SNP NAT2 rs1799931 G>A was in Hardy–Weinberg equilibrium in both groups. The wild-type GG genotype was the most frequent in both groups. The heterozygous GA genotype was found with a very low frequency only in the control group, while the homozygous mutated AA was not observed.

The distributions of the UGT1A1 genotypes were in Hardy–Weinberg equilibrium in both groups. Results showed that the heterozygous genotype was present only in controls but not in ASD patients, with a protective effect. We found the mutated homozygous genotype only in individuals with ASD, with an OR of 24 times.

Genotyping results for the variants *A (Ile105Val) and *B (A114V) of GSTP1, deleted/null of GSTM1, and deleted/null of GSTT1 (deletion) showed that the genotype distributions were in agreement with Hardy–Weinberg equilibrium in both groups.

The high diversity of the genotypes determined a fragmentation of the results; the different haplotypes of GSTP1, resulting from the different combinations of the alleles *A (I105V) and *B (A114V), were grouped and their distribution was analyzed as a sum of the frequencies.

The results showed a significantly higher frequency of the wild-type genotype in the control group than in ASD patients (Table 3). 

Instead, mutated heterozygous and homozygous genotypes were more frequent in ASD patients than in controls, even if these differences were not significant likely because of the small number of subjects included in the subgroups corresponding to different GSTP1 genotypes (Table 3). The sum of mutated genotypes I105V/A114A, V105V/A114A, I105V/A114V, and I105I/A114V, had a higher frequency in patients, but these differences were not significant. The genotype I105V/V114V was not found in both groups examined.

Individuals with I105V/A114V genotype had a higher frequency in controls, but also in this case there were not statistically significant differences.

Analysis of the deletion *M1 for GST revealed no differences between controls and patients.

On the contrary, analysis of the deletion *T1 for GST revealed important statistically significant differences between controls and patients. In fact, the deleted genotype was more frequent in the latter group, and it was associated with the pathology with an OR of 2.5. In controls, the not deleted genotype was more common.

The frequency of subjects that had both deletions was higher in patients than controls, and this difference was statistically significant.

### 2.3. Evaluation of Oxidative Stress Markers

The measurements of oxidative stress markers, namely dROMs, AOPP and BAP, showed that the mean values of concentration in affected individuals were higher than in controls (Table 3). These data indicated a mild oxidative stress status. These differences were significant for AOPP, with a *p* value <0.0001. dROMs and BAPs values were similar in controls and patients.

We further analyzed the variability of oxidative stress markers in ASD patients having different genotypes. In particular, the study cohort was divided in three groups: (1) carriers of SOD2 wild-type genotype and several other SNPs among those examined in this study (total number of mutated alleles = 2–9), (2) carriers of SOD2 A16V heterozygous genotypes and several other SNPs among those examined in this study (total number of mutated alleles: 2–6); (3) carriers of SOD2 A16V mutated genotype and several other SNPs among those examined in this study (total number of mutated alleles = 2–6). In the comparison between the different groups, the heterozygous patients showed lower mean levels of AOPP and dROMs, and the highest mean BAP. On the contrary, the homozygous group showed the highest mean levels of AOPP and dROMs, while the lowest mean BAP was found in the wild-type group (Table 4). However, a significant difference was only observed for BAP levels between the wild-type patients and patients carrying either the SOD2 A16V heterozygous genotype (*p* = 0.006) or mutated homozygous genotype (*p* = 0.004).

### 2.4. Comet Assay DNA Damage

**Figure 1:**
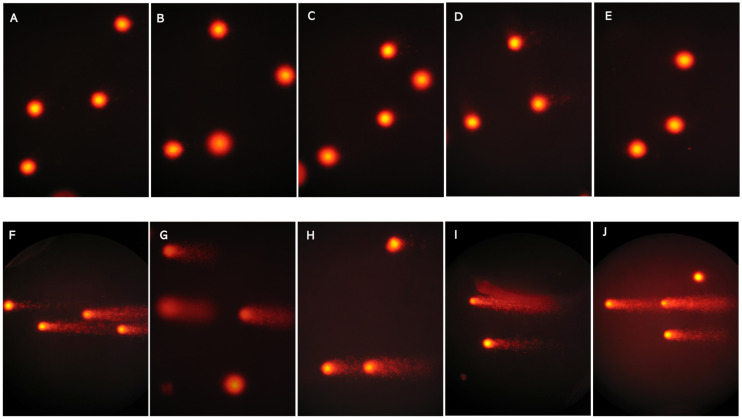
Representative images of the lymphocytes analyzed by the SCGE (Comet assay). (**A**–**E**), microscope image in 5 control subjects; (**F**–**J**) SCGE microscope image in 5 ASD patients.

The microscopical observation of lymphocytes subjected to Comet assay revealed a higher level of DNA damage in nuclei of lymphocytes isolated from ASD patients compared with healthy controls (Figure 1).

Moreover, the measurement of comet parameters showed that there were significant differences between healthy controls and individuals with ASD (Table 5).

## 3. Discussion

ASD is a group of neurodevelopmental disorders with heterogeneous clinical manifestation and various degrees of severity. Despite the large research on the matter, a clear etiology has not been identified and currently the diagnosis is based on clinical observation of the symptoms [1]. The impact of the genetic inheritance on ASD etiopathogenesis has long been recognized, since Autism shows a 90% heritability in monozygotic twins but only 0–10% in dizygotic twins, and several monogenic disorders present with neurodevelopmental impairment and autistic traits [3,34]. However, only in <1% of cases a pathogenic variant is identified, and the majority of ASD cases are not the consequence of a simple single gene or chromosomal disorder. Therefore, ASD is considered a multifactorial disease with multiple genes involvement and epigenetic influence [3,17]. Moreover, given the lack of evidence on the causative mechanisms of ASD, current pharmacological treatments address the associated symptoms of ASD and not the core manifestations, with the possibility of developing several and significant adverse effects [35].

Recently, research on the mechanisms involved in the occurrence of ASD focused on environmental, metabolic, and immune contribution, taking into account the additive and potentially synergistic effects of these risk factors [36]. To date, several neurotoxic and inflammatory agents have been investigated, suggesting that gestational exposure to xenobiotics may increase the risk of an ASD diagnosis in the offspring. On the contrary, some substances pertaining to the detoxification pathways (i.e., folic acid) represent protective factors [17]. In this light, some authors proposed oxidative stress as the linking mechanism, given that response to oxidative stress is genetic determined but also influenced by environmental factors [36,37].

Oxidative stress-related biomarkers have been previously linked to neurodevelopmental impairments associated with ASD and abnormalities of the brain that have a higher prevalence in autistic people [38,39,40,41,42]. Moreover, numerous studies identified an imbalance between prooxidant and antioxidants in individuals with ASD [38]. As a matter of fact, oxidative stress during the pregnancy has been related to several postnatal diseases of the children [43]. The human brain is particularly vulnerable to oxidative damage since it represents only the 2% of the body weight but it consumes approximately 20% of the basal oxygen; this is even truer in autistic children, that have an increased brain volume in comparison to healthy subjects [44,45,46]. Moreover, it has high energy demands and it is rich in unsaturated fatty acids, which are especially susceptible to oxidation: indeed, the brain has a higher oxidizing capacity in comparison to the antioxidant resources and the produced oxidative stress determines a damage to biomolecules (i.e., lipids, proteins, and nucleic acids), inducing profound consequences in the developing central nervous system [28,37,44]. Furthermore, the cerebellum represents a region of the central nervous system that has the highest number of neurons and it is selectively vulnerable to environmental insults (i.e., hypoxia-ischemia, excitotoxicity, viral infections, vitamin deficiencies, heavy metals exposure, bilirubin, etc.) and, particularly, to oxidative stress [46,47,48]. The latter affects especially large neurons, inducing loss of Purkinje cells and atrophy of the cerebellar folia, that have been consistently involved in the pathogenesis of ASD [46].

In this study, we evaluated and compared the genetic predisposition to oxidative response and its relationship with altered oxidative stress markers in individuals with ASD and healthy controls. We investigated the oxidative stress markers like dROMs, AOPP and BAP, which confirmed a mild oxidative stress status in the autistic patients in comparison to the controls. In particular, the AOPP showed a statistically significant difference, accordingly to previous studies [45,49,50]. Especially, Nasrallah and Alzeer proved a significant difference between the ASD subjects and the not related typically development (TD) controls, though this difference was not statistically relevant when comparing individuals with ASD to their siblings [45]. This finding suggests a genetic predisposition to oxidative stress that is shared by patients and their siblings, representing the substrate on which environmental agents may intervene to determine the disorder.

Regarding the SNPs involved in the oxidative stress response, most of the individuals in our ASD group presented a polymorphism in the SOD2 gene; particularly, most of our patients showed the heterozygous genotype causing a Ala16Val amino acid substitution. SOD is considered the only antioxidant enzyme capable of detoxifying superoxide anion and a reduction in its activity may cause incomplete superoxide neutralization [38]. Reports in the literature are inconclusive about the role of SOD on the etiopathogenesis of ASD: when compared to healthy individuals, some studies found increased SOD activity, while other research groups refuted this data, reporting significantly lower activity of SOD in children with ASD; moreover, no significant difference among the groups was indicated by other works [51]. It is worthy of mention that a real comparison among the different studies is difficult to achieve, since in most of the literature SOD activity was reported irrespective of the isozyme subclass and considered in several biological samples (i.e., erythrocytes, platelets, or plasma) [28]. Furthermore, very few researchers investigated the SOD gene SNPs in the autistic population. Kovač and colleagues reported a statistically significant association of ASD with two variants of the SOD1 gene, while they found no significant difference in the SOD2 A16V polymorphism between the patients and the controls [52]. Conversely, Esparham and colleagues stated that the majority of individuals with ASD in their cohort showed a homozygous or heterozygous variant in the SOD2 A16V; however, the number of affected individuals examined in this research (n = 7) is very small and not compared with a control group [53]. In fact, in contrast with this latter research, most of the healthy individuals in our study presented homozygous TT polymorphism.

To better investigate the relationship between the SOD2 gene and the ASD etiopathogenesis, we analyzed the oxidative stress markers in individuals with ASD and controls with different SOD2 genotypes. Specifically, the heterozygous patients showed lower mean levels of AOPP and dROMs, and the highest mean BAP. On the contrary, the lowest mean BAP was found in the wild-type group and showed a significant difference with the patients carrying the SOD2 A16V heterozygous genotype and even in comparison with the heterozygous and homozygous groups taken together. In the literature, only a Japanese group evaluated the association between these markers of oxidative stress and the ASD: they detected higher levels of d-ROMs in ASD patients in comparison to TD individuals e proposed the BAP/d-ROMs ratio (antioxidant capacity) as a reliable biomarker of to assess oxidative stress in children with ASD [54,55].

Very few data are also reported in the literature regarding the catalase, with a reduction or an enhance in its enzymatic activity depending on the examined samples (i.e., erythrocytes or plasma and erythrocytes), or else no association with ASD [28,51,56]. Nevertheless, to the best of our knowledge, we could not find any study that investigated the association between CAT polymorphisms and ADS and, therefore, our work is the first study to detect a significant association with the SNP CAT -844 C>T, being the wild-type a protective factor for the pathology and the heterozygous genotype a 2 times higher risk factor for developing ASD. Moreover, according to the data reported by Manivasagam et al. (2020) that described altered enzymatic activities of catalase and glutathione peroxidase in autistic individuals, our results also showed a statistically significant higher number of GPx polymorphisms in the patients group [56]. Particularly, GPx is one of the most important antioxidant enzymes counteracting oxidative stress and the C>T substitution in the GPx gene has been previously related to other neuropsychiatric disorder (i.e., schizophrenia): Shao et al. (2020) [57] proved a significant difference between schizophrenic patients and controls, suggesting a protective role of the C-allele. Consistent with these data, we found the TT variant only in the ASD group, making this homozygous genotype a 23 times higher risk factor for developing ASD [57].

In the literature, there are controversial data regarding the association between PON1 polymorphisms and their association to ASD. However, in a recent systematic review Banhela and colleagues analyzed the association between paraoxonase-1 SNPs and neurobehavioural outcomes in children and concluded that, though polymorphic variants on the PON1 gene show great variability which may influence the activity and functionality of enzymes encoded by the gene, the polymorphisms 108T and Q192R are associated with adverse neurobehavioural development [58]. In our sample, the healthy individuals showed a significantly higher frequency of the wild-type Q192R polymorphism, and this genotype resulted in being protective against ASD, with a 2 times lower risk for the disease. On the contrary, the homozygous mutated genotype was only observed in ASD individuals and associated with a 31 times higher risk for the disease. Moreover, in a comparative study performed by Gaita and colleagues (2010), serum arylesterase activity in combination with PON1 SNPs rs705379 (C-108T) and rs662 (Q192R) was able to discriminate ASD patients from controls with elevated sensitivity and specificity [59]. Paraoxonase 1 is a multifunctional enzyme involved in oxidative stress and capable to hydrolyze several toxic compounds that have been considered risk factors for the neurodevelopment, such as organophosphorus insecticides and their active metabolites, as well as nerve agents [17,60]. Thus, the mutant genotype detected only in the ASD group is consistent with the higher risk of developing the pathology.

GST represents another important antioxidant defense mechanism by performing the detoxification of xenobiotics and inactivation of endogenous oxidative stress products [61]. Particularly, in our study, we considered the polymorphisms of the different isoforms GSTP1, GSTM1, and GSTT1, which have been reported to be associated with increased risk of developing ASD [61]. Although not significant because of the small group, individuals with ASD in our study showed a higher frequency of the mutated heterozygous and homozygous GSTP1 genotypes than in controls. When considering the Ile105Val polymorphism, the healthy subjects showed a significant difference compared to the autistic patients, giving to the GSTP1*Ile105 allele a protective action against the disease. This data supports the evidence reported by Morales and colleagues, who proposed the GSTP1 mutated polymorphism as a potential risk factor for cognitive impairment, especially in children exposed to xenobiotics during the pregnancy [62]. In fact, almost a patient on two with autism also shows intellectual disability and the literature agrees that these pathologies share a common genetic substrate [40]. Moreover, an increased risk of ASD was observed if a combined GSTM1-active and GSTP1*llelle genotype was present [63].

Considering the GSTM1 polymorphisms, our work did not show statistically significant difference between the two groups; on the contrary, the deletion *T1 for GST revealed a statistically significant difference in individuals with ASD compared to controls, outlining a 2.3 times higher risk of developing the disorder. In addition, when combined to the GSTM1 null genotype, the risk increased to 5 times. Very few studies investigated the role of the glutathione transferase isoforms SNPs in the etiopathogenesis of ASD. Nevertheless, GSTT1 has been previously associated with a higher risk of developing epilepsy, which constitutes an important comorbidity of ASD, while the deletion *M1 for GST has been link to worse adaptative functions in autistic children [61].

All these pieces of evidence taken together enforce the concept that the etiopathogenesis of ASD is to be searched in a gene-environment interaction, in which oxidative stress has a major role: in fact, proteins are main targets of the reactive oxygen species and their oxidation results in modification of their structure and function, often in an irreparable manner [37,45].

ASD patients’ treatment requires a multimodal approach, including behavioral, educational, and pharmacological approaches; previous studies reported a use of medications in autistic children up to 83% [64]. In this light, evaluation of the cytochrome P450 enzyme system SNPs is considerably intriguing, since 30-50% of the ASD patients do not show an adequate response to treatment or incur in adverse events [65]. Particularly, the genotype distributions for CYP2C9*2 and *3 polymorphisms represent interesting data, since, to the best of our knowledge, this is the first study to investigate their association with ASD. In fact, the CYP2C9 is responsible for the metabolism of several drugs, and the mutated homozygous CYP2C9*2 genotype was related to refractory epilepsy and the homozygous *3 polymorphism has been related to lower levels of anti-seizures medications [66,67]. Our study found that the mutated heterozygous *1/*3 genotype was significantly more frequent in the ASD group and associated with a 20 times increased risk for the disease. This evidence has an impact on pharmacological treatment that is of the utmost importance in consideration that autistic patients have an increased incidence of epileptic seizures and EEG abnormalities in comparison to healthy population, the latter of which are also associated with the severity of cognitive and behavioral disturbances [68].

Although the distribution for the CYP2D6 polymorphisms showed higher frequency of the *1/*1 genotype both in the ASD patients and in the controls, we found a significant difference among the groups, that resulted in a reduction in the risk for the disease by 53 times. The heterozygous *1/*41 genotype was higher in the affected individuals, and it was associated with a 44 times increased risk for the disease, while the *41 mutated allele was only found in ASD group. CYP2D6 is responsible for the metabolism of many psychiatric medications and especially risperidone, which is the most frequently prescribed antipsychotic drug to children and adolescents [69]. As already mentioned, a modification in the function of the CYP may induce different responses to treatment: poor metabolizers are at higher risk of adverse events, while ultra-rapid metabolizers represent the group that may incur in therapeutic failure [70]. The *41 allele has been previously associated with reduced activity of the CYP2D6, causing a higher risk of risperidone-induced hyperprolactinemia and other side effects in the poor metabolizers [70,71,72].

Lastly, UGT1A1 has also been linked to risperidone-induced hyperprolactinemia, though the exact mechanism for this association is not elucidated yet [72]. In our study, the heterozygous SNP *1/*6 was only reported in controls while the homozygous genotype *6/*6 was identified only in ASD patients, with a 24 times increased risk of developing the disease. However, in a recent study, Horinouchi and colleagues did not find a significant difference in the frequency of the SNP between 79 ASD patients and the healthy control population, nor association between ASD symptoms and this polymorphism [73].

Overall, we found significant associations between ASD and several polymorphisms of genes involved in the detoxification and the response to oxidative stress. This evidence enforces the abundant literature linking oxidative stress to ASD etiology [29,38,46,61]. However, an important role is also played by environmental factors but the literature explored the interaction between xenobiotics and genetic predisposition is still scarce [17,37,74].

It is important to emphasize that systemic oxidative stress and inflammation have been described in other psychiatric disorders, including schizophrenia, bipolar disorders, and anxiety disorders, and are related to homeostatic dysregulation [75,76]. Several factors can alter the antioxidant systems, such as age, sex, medications, and lifestyle but further studies are needed to determine whether there are specific characteristics/alterations for different psychiatric conditions through cross-diagnosis comparisons. Furthermore, while our study assessed key aspects of oxidative balance through dROMs, AOPP, BAP, and SNPs in enzymatic antioxidant genes, we acknowledge that it does not cover the full spectrum of oxidative stress pathways. Markers of lipid peroxidation and DNA oxidation could provide additional mechanistic insights. Future studies incorporating these measures would allow a more complete characterization of redox dysregulation.

Notably, children with ASD commonly exhibit selective eating behaviors that may result in deficiencies of essential nutrients, insufficient fiber and fluid intake, and consequent gastrointestinal symptoms such as constipation or diarrhea [77]. Positive results have been obtained with gluten-free, soy-free, and dairy-free diets, but making dietary changes requires first being tested for celiac disease [78]. According to some research, vitamin and mineral supplements, due to their antioxidant effects, can influence various metabolic and nutritional alterations [79]. Nutraceutical compounds such as carotenoids, polyphenols, and omega-3 fatty acids have shown promise in mitigating autistic symptoms and comorbidities through their antioxidant effects [80,81]. Similar benefits have been observed with selected antioxidants (vitamin E, vitamin C, N-acetylcysteine) or diets rich in nutraceuticals, which may act additively or synergistically [80,82]. As individuals with ASD present with both increased oxidative stress and nutritional deficiencies, nutritional assessment represents an important aspect of clinical care. Usually, an ASD diagnosis is not achieved before 2 years of age, though the disruptive factors implicated in the etiopathogenesis of the disorder are known to intervene since the early stages of neurodevelopment, already during the pregnancy [26,29,38,61]. Nevertheless, data in the literature indicate that early detection of developmental disorders improves substantially the outcome of the child, since the intervention may be performed during the peak of brain plasticity [83]. However, further studies need to be conducted to prove the effectiveness of antioxidant supplementation in improving symptoms in children with ASD. When treating patients with ASD, it is also essential to understand the potential impact of diet on improving patients’ overall well-being.

## 4. Materials and Methods

### 4.1. Study Cohorts

One hundred six (83 M, 23 F; 7.9 ± 3.2 years) patients with ASD diagnosis were randomly recruited among those attending the Unit of Child Neurology and Psychiatry at Polyclinic Hospital University in Messina (Italy).

All the recruited subjects were diagnosed as affected by ASD on the basis of a clinical evaluation of symptoms. In particular, subjects fulfilling DSM-V (fifth edition of Diagnostic and Statistical Manual of Mental Disorders) diagnostic criteria [1] for ASD were screened for non-syndromic autism using MRI (Magnetic Resonance Imaging), EEG (Electroencephalogram), audiometry, urinary amino acids and organic acids, cytogenetic and fragile-X testing. Patients with dysmorphic features were excluded even in the absence of detectable cytogenetic alterations. Patients with sporadic seizures (i.e., <1 every 6 months) were included; patients with frequent seizures or focal neurological deficits were excluded. Informed written consent to blood sampling and anamnestic data collection was obtained from parents of all recruited ASD subjects and from healthy subjects. The study protocol was approved by the Ethics Committee of Polyclinic Hospital University (protocol number 89/16, date 10 November 2016) and was carried out in accordance with the ethical standards of Helsinki declaration and its later amendments.

A cohort of ninety healthy subjects (63 M, 27 F; 21.2 ± 1.8 years), recruited for previous studies (protocol number 51/17 date 10 July 2017; protocol number 57/21, date 8 February 2021; protocol number 24/22 date 15 February 2022), was included for comparison of genotype frequencies in the local population.

### 4.2. Genotyping by Real-Time PCR-Based Allelic Discrimination

Genomic DNA was isolated from peripheral blood lymphocytes by Gentra Pure Gene DNA Purification System (Qiagen, Milan, Italy), according to the manufacturer’s protocol. DNA quantification was carried out by spectrophotometric methods, and DNA integrity was checked by agarose gel electrophoresis. Genotyping of ASD patients and control subjects for CAT -844T>C (C_7618104_10); GPx1 rs1800668 (C_7912052_40); PON1 Q192R (C_2548962_20); SOD2 A16V (C_8709053_10); GSTP variant 1A (Ile105Val) (C_3237198_20); GSTP variant 1B (Ala115Val) (C_104961520); CYP2C9*2 (C_25625805_10); CYP2C9*3 (C_27104892_10); CYP2C19*2 (C_25986767_70); CYP2D6*41 (C_34816116_20), AHR Arg554Lys (C_11170747_20), NAT1 (C_1204334_50), NAT2 (C_572770_20) and UGT1A1*6 (C_2307598_20) was carried out by Real-time PCR allelic discrimination using pre-designed TaqMan SNP Genotyping assays available from Applied Biosystems (Applera Italia, Monza, Italy).

Genotyping reactions were set up in a 96-well plate on a 7500HT Fast Real-Time PCR System (Applied Biosystems, Foster City, CA) and were carried out in a final volume of 10 μL containing 1× TaqMan Genotyping Master Mix, 1× TaqMan-specific assay, and 10 ng genomic DNA, using thermal cycling conditions suggested by manufacturer’s protocols.

### 4.3. Genotyping by PCR and Electrophoresis of GST Deletion Variants

The GSTM1 and GSTT1 gene deletions were examined by PCR and electrophoresis, as previously reported [84]. Briefly, amplification consisted of a denaturation step at 94 °C for 4 min, annealing at 94 °C for 30 s, 64 °C for 30 s, and 72 °C for 30 s for 30 cycles, and polymerization at 72 °C for 7 min for 1 cycle. Forward and reverse primers for GSTM1 were GAACTCCCTGAAAAGCTAAAGC and GTTGGGCTCAAATATACGGTGG and for GSTT1 were TTCCTTACTGGTCCTCACATCTC and TCACCGGATCATGGCCAGCA. The GSTM1 fragment has a length of 215 bp; the GSTT1 fragment has a length of 489 bp. Human albumin was used as the positive control with forward and reverse primers of GCCCTCTGCTAACAAGTCCTAC and GCCCTAAAAAGAAAATCGCCAATC and has a fragment of 351 bp. Each 50-μL PCR reaction contained 5 μL of 10× PCR buffer, 5 μL of 25 mM MgCl2, 1 μL of 10 mM deoxynucleotides (ACGT), 1 μL of each primer (forward and reverse primers GSTT1 and GSTM1), 0.5 μL of forward and reverse primers for human albumin, 26.36 μL of ddH2O, 0.44 μL Taq, 1.76 μL TaqStart buffer, 0.44 μL TaqStart antibody, and 5 μL of unknown sample DNA. PCR products were visualized by agarose gel (2%) electrophoresis. This assay does not distinguish between heterozygous and homozygous presence of either gene; therefore, patients are designated either “homozygous null” or “heterozygous/homozygous present.” All plates contained blinded duplicates and positive and negative controls for quality control.

### 4.4. Assessment of Oxidative Stress Levels

In order to evaluate oxidative stress levels in ASD patients having different genetic backgrounds, the measurement of the derivatives of reactive oxygen metabolites (dROMs), the Biological Antioxidant Potential (BAP), and the advanced oxidation protein products (AOPP) were performed. We selected dROMs, AOPP, and BAP to capture complementary aspects of systemic oxidative balance, reflecting global oxidative burden, protein oxidation, and overall non-enzymatic antioxidant capacity, respectively.

The dROM test and BAP test were carried out according to the manufacturer’s protocol (Diacron International, Grosseto, Italy).

dROMs reflect the amount of organic hydroperoxides that are related to the free radicals from which they are formed. The results are expressed in arbitrary units (Carratelli units), one unit of which corresponds to 0.8 mg/L of hydrogen peroxide.

The BAP test provides an estimate of the global antioxidant capacity of blood plasma, measured as its reducing potential against ferric ions. The results are expressed in µmol/L of the reduced ferric ions [85].

The measurement of AOPP serum levels was performed using a colorimetric method involving chloramine T as a standard, as described by Alazoglu and coworkers (2013) [86]. The assay was carried out in a 96-well microplate and sample absorbance was determined using a Sunrise microplate reader (Tecan Italia, Vernusco sul Naviglio, Milan, Italy).

### 4.5. Assessment of Oxidative DNA Damage by Single Cell Gel Electrophoresis (Comet Assay)

The single cell gel electrophoresis (SCGE), is a sensitive technique for quantifying and analyzing oxidative DNA damage in individual cells [87,88].

Lymphocytes were isolated from 3 mL of blood samples through the use of Histopaque 1077 (Sigma-Aldrich, Milan, Italy), resuspended with Low Melting Agar and spotted on a slide. The slides were placed in an electrophoretic pan and subjected to electrophoresis at 300 mA for 30 min in the dark. Ethidium bromide, an intercalating agent for nucleic acid stain, was used as a dye for all slides. After 24 h samples were analyzed by the use of a Leica microscope and images were analyzed by CASP software 1.2.2., as reported by Gugliandolo and coworkers (2016) [89].

The parameters analyzed were Head Length, Tail Length, Comet Length, Head DNA and Tail DNA amount.

### 4.6. Statistical Analysis

Continuous variables were analyzed using unpaired Student’s *t*-test, while categorical variables and compliance of allele distribution to the Hardy–Weinberg equilibrium were evaluated by Fisher’s exact test.

Statistically different effects of gene polymorphisms on biochemical variables were evaluated by a one-way analysis of variance (ANOVA) followed by the Newman-Keuls post hoc test. Significant values were assessed according to *p* < 0.05.

All the analyses were carried out using IBM SPSS statistic v22.

## 5. Conclusions

Both genetic and environmental factors contribute to the onset of autism spectrum disorder, and an early recognition of ASD symptoms would help affected children develop adaptation skills, leading to better social integration. For this reason, further studies are required to better understand the mechanisms involved in the development of ASD and to discover reliable and precocious biomarkers of the disease in order to implement an early detection of the disorder and an intensive intervention.

## Figures and Tables

**Table 1:** Distribution of gene polymorphisms of antioxidant enzymes in ASD patients and healthy controls.

Genotype	ASD (*n* = 106) %	Controls (*n* = 90) %	*p*-Value	Odds Ratio (95% C.I.)
*SOD2 A16V*				
AA	33.02	21.11	0.0777	-
AV	36.79	26.67	0.1671	-
VV	30.19	52.22	0.0021	0.3956 (0.2202 to 0.7107)
*CAT -844C>T*				
CC	53.77	68.89	0.0398	0.5253 (0.2920 to 0.9453)
CT	46.23	31.11	0.0398	1.904 (1.058 to 3.425)
TT	-	-		-
*GPx1 rs180668 C>T*				
CC	54.81	75	0.0028	0.3924 (0.2118 to 0.7270)
CT	34.62	25	0.1569	-
TT	10.58	0	0.001	22.26 (1.292 to 383.6)
*PON Q192R T>C*				
TT	47.62	63.33	0.0471	0.5263 (0.2871 to 0.9649)
TC	38.10	36.67	0.8763	-
CC	14.29	0.00	0.0001	31.21 (1.816 to 536.4)

**Table 2:** Distribution of gene polymorphisms in phase I and II xenobiotic metabolism enzymes.

Genotype	ASD (*n* = 106) %	Controls (*n* = 90) %	*p*-Value	Odds Ratio (95% C.I.)
CYP2C9*2, *3 ^a^				
*1/1	73.3	84.4	0.0524	-
*1/*2	6.7	12.2	0.1058	-
*1/*3	18.3	1.1	0.0011	18.88 (2.236–159.4)
*2/*2	1.7	2.2	1	-
CYP2C19*2 ^b^				
*1/*1	73.91	80.0	0.445	-
*1/*2	21.74	16.7	0.4228	-
*2/*2	4.35	3.33	1	-
CYP2D6*41 ^c^				
*1/*1	77.5	100	<0.0001	0.01878 (0.001–0.340)
*1/*41	20	-	0.0002	44.4 (2.420–814.6)
*41/*41	2.5	-	0.2623	-
*AHR Arg254Lys* ^d^				
Arg/Arg	83.9	77.8	0.4109	-
Arg/Lys	16.1	16.7	1	-
Lys/Lys	-	5.5	0.0797	-
*NAT1* Arg187Gln ^f^				
Arg/Arg	97.26	96.67	1	-
Arg/Gln	2.74	3.33	1	-
Gln/Gln	-	-	-	-
*NAT2rs1799931 G>A* ^g^				
G/G	100	96.67	0.2466	-
G/A	-	3.33	0.2466	-
A/A	-	-	-	-
UGT1A1*6 G>A ^h^				
WT 1/1 G/G	89.09	85.56	0.6191	
HT *1/*6 G/A	-	14.44	0.0019	0.05172 (0.003–0.889)
MUT *6/*6 A/A	10.91	-	0.0025	23.77 (1.310–431.1)
*GSTP1 I105V/A114V*				
*A/*A	45.2	62.2	0.024	0.5025 (0.283–0.891)
*A/*B	34.5	28.9	0.5378	-
*B/*B	8.3	4.4	0.3888	-
*A/*C	4.8	2.2	0.4558	-
*A/*D	1.2	-	1	-
*B/*C	5.9	2.2	0.2923	-
*GSTM1 Null* (*0) ^e^				
*1/*1+*1/*0	58.8	53.3	0.1494	-
*0/*0	41.2	46.7	0.1494	-
*GSTT1 Null* (*0) ^e^				
*1/*1+*1/*0	64.7	80	0.0296	0.43 (0.210–0.878)
*0/*0	35.3	20	0.0296	2.326 (1.139–4.750)
*GSTM1 Null*/ *GSTT1 Null* ^e^				
(*1/*1+*1/*0)/ (*1/*1+*1/*0)	38.2	47	0.615	-
(*1/*1+*1/*0)/*0/*0	16.2	15.5	0.915	-
*0/*0/(*1/*1+*1/*0)	22.05	33.3	0.12	-
(*0/*0)/(*0/*0)	19.1	4.44	0.0032	5.000 (1.64–15.24)

Legend: ^a^ ASD examined were 60; ^b^ ASD examined were 70; ^c^ ASD examined were 40; ^d^ ASD examined were 62; ^e^ ASD examined were 68; ^f^ ASD examined were 73; ^g^ ASD examined were 84; ^h^ ASD examined were 55. GSTP1*A = I105/A114; GSTP1*B = V105/A114; GSTP1*C = V105/V114; GSTP1 *D =I105/V114.

**Table 3:** Assessment of oxidative stress marker in ASD patients and case controls.

Redox Marker	ASD (n = 106)	Controls (n = 90)
AOPP	329.1 ± 149.7 ***	74.9 ± 15.1
dROMs	327.1 ± 115.9	319.3 ± 50.1
BAP	2428.0 ± 1479.3	2128.4 ± 1820.6

Values are provided as mean ± standard deviation. The unpaired *t*-test was used to compare statistical differences between ASD and control subjects. *** *p* < 0.0001 significant difference in comparison to controls.

**Table 4:** Variability of oxidative stress markers in ASD patients having different genotypes.

	AOPP	dROMs	BAP
Group 1 (SOD2 Wt + 2-9 other SNPs) (n = 35)	312.2 ± 111.9	357.3 ± 107.0	1694.0 ± 1061.9
Group 2 (SOD2 Ht + 2-6 other SNPs) (n = 39)	303.0 ± 134.3	347.3 ± 101.6	3008.8 ± 1396.2 **
Group 3 (SOD2 Mut + 2-6 other SNPs) (n = 32)	378.4 ± 151.0	401.7 ± 160.4	2392.5 ±1248.5 **

Values are provided as mean ± standard deviation. Statistical differences between groups have been evaluated by one way ANOVA followed by the Newman-Keuls post hoc test. ** *p* < 0.01 significant differences in comparison with controls.

**Table 5:** Variability of Comet ^+^ cells and variability of comet parameters in ASD patients and controls.

Comet Parameters	ASD (n = 32)	Controls (n = 27)
% COMET + cells	55.4 ± 4.66 ***	4.5 ± 2.9
Head length	215.8 ± 15.3	209.5 ± 15.7
Tail length	64.5 ± 14.1 ***	36.6 ± 9.5
Comet length	274.0 ± 20.3 ***	252.5 ± 13.6
Head DNA	85.9 ± 4.5	112.4 ± 101.9
Tail DNA	14.04 ± 4.6 *	11.6 ± 4.1
Tail Moment	14.8 ± 9.8 *	9.3 ± 6.4
Olive Tail Moment	11.2 ± 6.2 ***	4.0 ± 2.4

Values are provided as mean ± standard deviation. The unpaired *t*-test was used to compare statistical differences between ASD and control subjects. * *p* < 0.05 and *** *p* < 0.001 significant differences in comparison with controls.

## Data Availability

The study’s original contributions are included in the article. Further details can be directed to the corresponding author.

## Abbreviations

The following abbreviations are used in this manuscript:

ASD	Autism spectrum disorder
GST	Glutathione S-transferase
dROMs	Reactive oxygen metabolites
BAP	Biological Antioxidant Potential
AOPP	Advanced oxidation protein products
GSH	Glutathione
GSSG	Glutathione disulfide
DSM	Diagnostic and Statistical Manual of Mental Disorders
MRI	Magnetic Resonance Imaging
EEG	Electroencephalogram
ADOS-2	Autism Diagnostic Observation Schedule—Second Edition
ADI-R	Autism Diagnostic Interview—Revised
CARS	Children Autism Rating Scales
VABS	Vineland Adaptive Behavior Scales
SCGE	Single cell gel electrophoresis
SNPs	Single nucleotide polymorphisms
OR	Odds Ratio
PON1	Paraoxonase 1
SOD	Superoxide Dismutase
CAT	Catalase
GPX1	Glutathione peroxidase 1
CYP	Cytochrome P450
AHR	Aryl hydrocarbon receptor
NATs	N-acetyl transferases
UGTs	UDP-glucuronosyl transferases
GSTs GWAS	Glutathione-S-transferases Genome-Wide Association Studies

## References

[1] American Psychiatric Association (2013). American Psychiatric Association DSM-5 Task Force Diagnostic and Statistical Manual of Mental Disorders: DSM-5.

[2] Maenner M.J., Shaw K.A., Bakian A.V., Bilder D.A., Durkin M.S., Esler A., Furnier S.M., Hallas L., Hall-Lande J., Hudson A. (2021). Prevalence and Characteristics of Autism Spectrum Disorder Among Children Aged 8 Years-Autism and Developmental Disabilities Monitoring Network, 11 Sites, United States, 2018. Morb. Mortal. Wkly. Rep. Surveill. Summ. Wash. DC 2002.

[3] Napolitano A., Schiavi S., La Rosa P., Rossi-Espagnet M.C., Petrillo S., Bottino F., Tagliente E., Longo D., Lupi E., Casula L. (2022). Sex Differences in Autism Spectrum Disorder: Diagnostic, Neurobiological, and Behavioral Features. Front. Psychiatry.

[4] Tick B., Bolton P., Happé F., Rutter M., Rijsdijk F. (2016). Heritability of Autism Spectrum Disorders: A Meta-Analysis of Twin Studies. J. Child Psychol. Psychiatry.

[5] Ronald A., Hoekstra R.A. (2011). Autism Spectrum Disorders and Autistic Traits: A Decade of New Twin Studies. Am. J. Med. Genet. Part B Neuropsychiatr. Genet. Off. Publ. Int. Soc. Psychiatr. Genet..

[6] Havdahl A., Niarchou M., Starnawska A., Uddin M., van der Merwe C., Warrier V. (2021). Genetic Contributions to Autism Spectrum Disorder. Psychol. Med..

[7] Lim H.K., Yoon J.H., Song M. (2022). Autism Spectrum Disorder Genes: Disease-Related Networks and Compensatory Strategies. Front. Mol. Neurosci..

[8] Genovese A., Butler M.G. (2023). The Autism Spectrum: Behavioral, Psychiatric and Genetic Associations. Genes.

[9] Chaste P., Leboyer M. (2012). Autism Risk Factors: Genes, Environment, and Gene-Environment Interactions. Dialogues Clin. Neurosci..

[10] Wang M., Zhang X., Zhong L., Zeng L., Li L., Yao P. (2025). Understanding Autism: Causes, Diagnosis, and Advancing Therapies. Brain Res. Bull..

[11] Klei L., Sanders S.J., Murtha M.T., Hus V., Lowe J.K., Willsey A.J., Moreno-De-Luca D., Yu T.W., Fombonne E., Geschwind D. (2012). Common Genetic Variants, Acting Additively, Are a Major Source of Risk for Autism. Mol. Autism.

[12] Grove J., Ripke S., Als T.D., Mattheisen M., Walters R.K., Won H., Pallesen J., Agerbo E., Andreassen O.A., Anney R. (2019). Identification of Common Genetic Risk Variants for Autism Spectrum Disorder. Nat. Genet..

[13] Bragg M., Chavarro J.E., Hamra G.B., Hart J.E., Tabb L.P., Weisskopf M.G., Volk H.E., Lyall K. (2022). Prenatal Diet as a Modifier of Environmental Risk Factors for Autism and Related Neurodevelopmental Outcomes. Curr. Environ. Health Rep..

[14] Doi M., Usui N., Shimada S. (2022). Prenatal Environment and Neurodevelopmental Disorders. Front. Endocrinol..

[15] Khogeer A.A., AboMansour I.S., Mohammed D.A. (2022). The Role of Genetics, Epigenetics, and the Environment in ASD: A Mini Review. Epigenomes.

[16] Mouat J.S., LaSalle J.M. (2022). The Promise of DNA Methylation in Understanding Multigenerational Factors in Autism Spectrum Disorders. Front. Genet..

[17] Volk H.E., Ames J.L., Chen A., Fallin M.D., Hertz-Picciotto I., Halladay A., Hirtz D., Lavin A., Ritz B., Zoeller T. (2022). Considering Toxic Chemicals in the Etiology of Autism. Pediatrics.

[18] Welch C., Mulligan K. (2022). Does Bisphenol A Confer Risk of Neurodevelopmental Disorders? What We Have Learned from Developmental Neurotoxicity Studies in Animal Models. Int. J. Mol. Sci..

[19] Rossignol D.A., Frye R.E. (2014). Evidence Linking Oxidative Stress, Mitochondrial Dysfunction, and Inflammation in the Brain of Individuals with Autism. Front. Physiol..

[20] Xu N., Li X., Zhong Y. (2015). Inflammatory Cytokines: Potential Biomarkers of Immunologic Dysfunction in Autism Spectrum Disorders. Mediators Inflamm..

[21] Pangrazzi L., Balasco L., Bozzi Y. (2020). Oxidative Stress and Immune System Dysfunction in Autism Spectrum Disorders. Int. J. Mol. Sci..

[22] Waligóra A., Waligóra S., Kozarska M., Damasiewicz-Bodzek A., Gorczyca P., Tyrpień-Golder K. (2019). Autism Spectrum Disorder (ASD)-Biomarkers of Oxidative Stress and Methylation and Transsulfuration Cycle. Psychiatr. Pol..

[23] Spoto G., Butera A., Albertini M.L., Consoli C., Ceraolo G., Nicotera A.G., Rosa G.D. (2025). The Ambiguous Role of Growth Factors in Autism: What Do We Really Know?. Int. J. Mol. Sci..

[24] Zawadzka A., Cieślik M., Adamczyk A. (2021). The Role of Maternal Immune Activation in the Pathogenesis of Autism: A Review of the Evidence, Proposed Mechanisms and Implications for Treatment. Int. J. Mol. Sci..

[25] McLellan J., Kim D.H.J., Bruce M., Ramirez-Celis A., Van de Water J. (2022). Maternal Immune Dysregulation and Autism-Understanding the Role of Cytokines, Chemokines and Autoantibodies. Front. Psychiatry.

[26] Manti S., Spoto G., Nicotera A.G., Di Rosa G., Piedimonte G. (2023). Impact of Respiratory Viral Infections during Pregnancy on the Neurological Outcomes of the Newborn: Current Knowledge. Front. Neurosci..

[27] Nishimura Y., Kanda Y., Sone H., Aoyama H. (2021). Oxidative Stress as a Common Key Event in Developmental Neurotoxicity. Oxid. Med. Cell. Longev..

[28] Frustaci A., Neri M., Cesario A., Adams J.B., Domenici E., Dalla Bernardina B., Bonassi S. (2012). Oxidative Stress-Related Biomarkers in Autism: Systematic Review and Meta-Analyses. Free Radic. Biol. Med..

[29] Liu X., Lin J., Zhang H., Khan N.U., Zhang J., Tang X., Cao X., Shen L. (2022). Oxidative Stress in Autism Spectrum Disorder-Current Progress of Mechanisms and Biomarkers. Front. Psychiatry.

[30] Thorsen M. (2020). Oxidative Stress, Metabolic and Mitochondrial Abnormalities Associated with Autism Spectrum Disorder. Prog. Mol. Biol. Transl. Sci..

[31] Gevezova M., Minchev D., Pacheva I., Sbirkov Y., Yordanova R., Timova E., Kotetarov V., Ivanov I., Sarafian V. (2021). Cellular Bioenergetic and Metabolic Changes in Patients with Autism Spectrum Disorder. Curr. Top. Med. Chem..

[32] Bjørklund G., Tinkov A.A., Hosnedlová B., Kizek R., Ajsuvakova O.P., Chirumbolo S., Skalnaya M.G., Peana M., Dadar M., El-Ansary A. (2020). The Role of Glutathione Redox Imbalance in Autism Spectrum Disorder: A Review. Free Radic. Biol. Med..

[33] Iyanagi T. (2007). Molecular Mechanism of Phase I and Phase II Drug-Metabolizing Enzymes: Implications for Detoxification. Int. Rev. Cytol..

[34] Spoto G., Valentini G., Saia M.C., Butera A., Amore G., Salpietro V., Nicotera A.G., Di Rosa G. (2022). Synaptopathies in Developmental and Epileptic Encephalopathies: A Focus on Pre-Synaptic Dysfunction. Front. Neurol..

[35] Frye R.E., Rossignol D.A. (2014). Treatments for Biomedical Abnormalities Associated with Autism Spectrum Disorder. Front. Pediatr..

[36] Kałużna-Czaplińska J., Jóźwik-Pruska J. (2016). Chromatographic and Mass Spectrometric Techniques in Studies on Oxidative Stress in Autism. J. Chromatogr. B Analyt. Technol. Biomed. Life. Sci..

[37] Tanner S., Thomson S., Drummond K., O’Hely M., Symeonides C., Mansell T., Saffery R., Sly P.D., Collier F., Burgner D. (2022). A Pathway-Based Genetic Score for Oxidative Stress: An Indicator of Host Vulnerability to Phthalate-Associated Adverse Neurodevelopment. Antioxid. Basel Switz..

[38] Chen L., Shi X.-J., Liu H., Mao X., Gui L.-N., Wang H., Cheng Y. (2021). Oxidative Stress Marker Aberrations in Children with Autism Spectrum Disorder: A Systematic Review and Meta-Analysis of 87 Studies (N = 9109). Transl. Psychiatry.

[39] Di Rosa G., Lenzo P., Parisi E., Neri M., Guerrera S., Nicotera A., Alibrandi A., Germanò E., Caccamo D., Spanò M. (2013). Role of Plasma Homocysteine Levels and MTHFR Polymorphisms on IQ Scores in Children and Young Adults with Epilepsy Treated with Antiepileptic Drugs. Epilepsy Behav. EB.

[40] Postorino V., Fatta L.M., Sanges V., Giovagnoli G., De Peppo L., Vicari S., Mazzone L. (2016). Intellectual Disability in Autism Spectrum Disorder: Investigation of Prevalence in an Italian Sample of Children and Adolescents. Res. Dev. Disabil..

[41] Zhang M., Hu X., Jiao J., Yuan D., Li S., Luo T., Wang M., Situ M., Sun X., Huang Y. (2022). Brain White Matter Microstructure Abnormalities in Children with Optimal Outcome from Autism: A Four-Year Follow-up Study. Sci. Rep..

[42] Marseglia L.M., Nicotera A., Salpietro V., Giaimo E., Cardile G., Bonsignore M., Alibrandi A., Caccamo D., Manti S., D’Angelo G. (2015). Hyperhomocysteinemia and MTHFR Polymorphisms as Antenatal Risk Factors of White Matter Abnormalities in Two Cohorts of Late Preterm and Full Term Newborns. Oxid. Med. Cell. Longev..

[43] Cannavò L., Perrone S., Viola V., Marseglia L., Di Rosa G., Gitto E. (2021). Oxidative Stress and Respiratory Diseases in Preterm Newborns. Int. J. Mol. Sci..

[44] Usui N., Kobayashi H., Shimada S. (2023). Neuroinflammation and Oxidative Stress in the Pathogenesis of Autism Spectrum Disorder. Int. J. Mol. Sci..

[45] Nasrallah O., Alzeer S. (2022). Measuring Some Oxidative Stress Biomarkers in Autistic Syrian Children and Their Siblings: A Case-Control Study. Biomark. Insights.

[46] Kern J.K., Jones A.M. (2006). Evidence of Toxicity, Oxidative Stress, and Neuronal Insult in Autism. J. Toxicol. Environ. Health B Crit. Rev..

[47] Amore G., Spoto G., Ieni A., Vetri L., Quatrosi G., Di Rosa G., Nicotera A.G. (2021). A Focus on the Cerebellum: From Embryogenesis to an Age-Related Clinical Perspective. Front. Syst. Neurosci..

[48] Spoto G., Amore G., Vetri L., Quatrosi G., Cafeo A., Gitto E., Nicotera A.G., Di Rosa G. (2021). Cerebellum and Prematurity: A Complex Interplay Between Disruptive and Dysmaturational Events. Front. Syst. Neurosci..

[49] Essa M.M., Guillemin G.J., Waly M.I., Al-Sharbati M.M., Al-Farsi Y.M., Hakkim F.L., Ali A., Al-Shafaee M.S. (2012). Increased Markers of Oxidative Stress in Autistic Children of the Sultanate of Oman. Biol. Trace Elem. Res..

[50] Ahmad T.Y., Tawfeeq F., Alameen S. (2013). Biochemical Studies of Autism Spectrum Disorder Patients in Mosul City. Res. J. Chem. Sci..

[51] Yenkoyan K., Harutyunyan H., Harutyunyan A. (2018). A Certain Role of SOD/CAT Imbalance in Pathogenesis of Autism Spectrum Disorders. Free Radic. Biol. Med..

[52] Kovač J., Macedoni Lukšič M., Trebušak Podkrajšek K., Klančar G., Battelino T. (2014). Rare Single Nucleotide Polymorphisms in the Regulatory Regions of the Superoxide Dismutase Genes in Autism Spectrum Disorder. Autism Res. Off. J. Int. Soc. Autism Res..

[53] Esparham A.E., Smith T., Belmont J.M., Haden M., Wagner L.E., Evans R.G., Drisko J.A. (2015). Nutritional and Metabolic Biomarkers in Autism Spectrum Disorders: An Exploratory Study. Integr. Med. Encinitas Calif.

[54] Morimoto M., Hashimoto T., Tsuda Y., Nakatsu T., Kitaoka T., Kyotani S. (2020). Assessment of Oxidative Stress in Autism Spectrum Disorder Using Reactive Oxygen Metabolites and Biological Antioxidant Potential. PLoS ONE.

[55] Kitaoka T., Morimoto M., Hashimoto T., Tsuda Y., Nakatsu T., Kyotani S. (2020). Evaluation of the Efficacy of Drug Treatment Based on Measurement of the Oxidative Stress, Using Reactive Oxygen Metabolites and Biological Antioxidant Potential, in Children with Autism Spectrum Disorder and Attention Deficit Hyperactivity Disorder. J. Pharm. Health Care Sci..

[56] Manivasagam T., Arunadevi S., Essa M.M., SaravanaBabu C., Borah A., Thenmozhi A.J., Qoronfleh M.W. (2020). Role of Oxidative Stress and Antioxidants in Autism. Adv. Neurobiol..

[57] Shao X., Yan C., Sun D., Fu C., Tian C., Duan L., Zhu G. (2020). Association Between Glutathione Peroxidase-1 (GPx-1) Polymorphisms and Schizophrenia in the Chinese Han Population. Neuropsychiatr. Dis. Treat..

[58] Banhela N., Naidoo P., Naidoo S. (2020). Association between Pesticide Exposure and Paraoxonase-1 (PON1) Polymorphisms, and Neurobehavioural Outcomes in Children: A Systematic Review. Syst. Rev..

[59] Gaita L., Manzi B., Sacco R., Lintas C., Altieri L., Lombardi F., Pawlowski T.L., Redman M., Craig D.W., Huentelman M.J. (2010). Decreased Serum Arylesterase Activity in Autism Spectrum Disorders. Psychiatry Res..

[60] Costa L.G., Giordano G., Cole T.B., Marsillach J., Furlong C.E. (2013). Paraoxonase 1 (PON1) as a Genetic Determinant of Susceptibility to Organophosphate Toxicity. Toxicology.

[61] Mandic-Maravic V., Mitkovic-Voncina M., Pljesa-Ercegovac M., Savic-Radojevic A., Djordjevic M., Ercegovac M., Pekmezovic T., Simic T., Pejovic-Milovancevic M. (2021). Glutathione S-Transferase Polymorphisms and Clinical Characteristics in Autism Spectrum Disorders. Front. Psychiatry.

[62] Morales E., Sunyer J., Castro-Giner F., Estivill X., Julvez J., Ribas-Fitó N., Torrent M., Grimalt J.O., de Cid R. (2008). Influence of Glutathione S-Transferase Polymorphisms on Cognitive Functioning Effects Induced by p,p’-DDT among Preschoolers. Environ. Health Perspect..

[63] Mandic-Maravic V., Coric V., Mitkovic-Voncina M., Djordjevic M., Savic-Radojevic A., Ercegovac M., Matic M., Simic T., Lecic-Tosevski D., Toskovic O. (2019). Interaction of Glutathione S-Transferase Polymorphisms and Tobacco Smoking during Pregnancy in Susceptibility to Autism Spectrum Disorders. Sci. Rep..

[64] Lamberti M., Siracusano R., Italiano D., Alosi N., Cucinotta F., Di Rosa G., Germanò E., Spina E., Gagliano A. (2016). Head-to-Head Comparison of Aripiprazole and Risperidone in the Treatment of ADHD Symptoms in Children with Autistic Spectrum Disorder and ADHD: A Pilot, Open-Label, Randomized Controlled Study. Paediatr. Drugs.

[65] Arranz M.J., Salazar J., Bote V., Artigas-Baleri A., Serra-LLovich A., Triviño E., Roige J., Lombardia C., Cancino M., Hernandez M. (2022). Pharmacogenetic Interventions Improve the Clinical Outcome of Treatment-Resistant Autistic Spectrum Disorder Sufferers. Pharmaceutics.

[66] Eltalal S., El Ayouty M., El-Said A., Wahba Y. (2021). CYP2C9 (*2&*3) and CYP2C19 (*2&*3) Polymorphisms among Children with Nonlesional Epilepsy: A Single-Center Study. Acta Neurol. Belg..

[67] Wu X., Dong W., Li H., Yang X., Jin Y., Zhang Z., Jiang Y. (2021). CYP2C9*3/*3 Gene Expression Affects the Total and Free Concentrations of Valproic Acid in Pediatric Patients with Epilepsy. Pharmacogenomics Pers. Med..

[68] Nicotera A.G., Hagerman R.J., Catania M.V., Buono S., Di Nuovo S., Liprino E.M., Stracuzzi E., Giusto S., Di Vita G., Musumeci S.A. (2019). EEG Abnormalities as a Neurophysiological Biomarker of Severity in Autism Spectrum Disorder: A Pilot Cohort Study. J. Autism Dev. Disord..

[69] Kloosterboer S.M., de Winter B.C.M., Reichart C.G., Kouijzer M.E.J., de Kroon M.M.J., van Daalen E., Ester W.A., Rieken R., Dieleman G.C., van Altena D. (2021). Risperidone Plasma Concentrations Are Associated with Side Effects and Effectiveness in Children and Adolescents with Autism Spectrum Disorder. Br. J. Clin. Pharmacol..

[70] Youngster I., Zachor D.A., Gabis L.V., Bar-Chaim A., Benveniste-Levkovitz P., Britzi M., Soback S., Ziv-Baran T., Berkovitch M. (2014). CYP2D6 Genotyping in Paediatric Patients with Autism Treated with Risperidone: A Preliminary Cohort Study. Dev. Med. Child Neurol..

[71] Vanwong N., Ngamsamut N., Medhasi S., Puangpetch A., Chamnanphon M., Tan-Kam T., Hongkaew Y., Limsila P., Sukasem C. (2017). Impact of CYP2D6 Polymorphism on Steady-State Plasma Levels of Risperidone and 9-Hydroxyrisperidone in Thai Children and Adolescents with Autism Spectrum Disorder. J. Child Adolesc. Psychopharmacol..

[72] Biswas M., Vanwong N., Sukasem C. (2022). Pharmacogenomics in Clinical Practice to Prevent Risperidone-Induced Hyperprolactinemia in Autism Spectrum Disorder. Pharmacogenomics.

[73] Horinouchi T., Maeyama K., Nagai M., Mizobuchi M., Takagi Y., Okada Y., Kato T., Nishimura M., Kawasaki Y., Yoshioka M. (2022). Genetic Analysis of UGT1A1 Polymorphisms Using Preserved Dried Umbilical Cord for Assessing the Potential of Neonatal Jaundice as a Risk Factor for Autism Spectrum Disorder in Children. J. Autism Dev. Disord..

[74] Spoto G., Di Rosa G., Nicotera A.G. (2024). The Impact of Genetics on Cognition: Insights into Cognitive Disorders and Single Nucleotide Polymorphisms. J. Pers. Med..

[75] Jorgensen A., Baago I.B., Rygner Z., Jorgensen M.B., Andersen P.K., Kessing L.V., Poulsen H.E. (2022). Association of Oxidative Stress-Induced Nucleic Acid Damage With Psychiatric Disorders in Adults: A Systematic Review and Meta-Analysis. JAMA Psychiatry.

[76] Steullet P. (2024). Editorial for the Special Issue “Oxidative Stress, Inflammation and Antioxidant Defense System in Psychiatric Disorders” in Antioxidants (2022–2023). Antioxid. Basel Switz..

[77] Schröder S.S., Danner U.N., Spek A.A., van Elburg A.A. (2022). Problematic Eating Behaviours of Autistic Women-A Scoping Review. Eur. Eat. Disord. Rev. J. Eat. Disord. Assoc..

[78] Al-Beltagi M., Saeed N.K., Bediwy A.S., Elbeltagi R., Alhawamdeh R. (2023). Role of Gastrointestinal Health in Managing Children with Autism Spectrum Disorder. World J. Clin. Pediatr..

[79] Taha Z., Abdalhai K.A. (2021). A Review of the Efficacy of the Dietary Intervention in Autism Spectrum Disorder. Open Access Maced. J. Med. Sci..

[80] Pangrazzi L., Balasco L., Bozzi Y. (2020). Natural Antioxidants: A Novel Therapeutic Approach to Autism Spectrum Disorders?. Antioxid. Basel Switz..

[81] Albertini M.L., Spoto G., Ceraolo G., Fichera M.F., Consoli C., Nicotera A.G., Di Rosa G. (2025). Sleep Disorders in Children with Autism Spectrum Disorder: Developmental Impact and Intervention Strategies. Brain Sci..

[82] Colak H., Sariyer E.T., Nogay N.H. (2023). The Effect of Nutritional Interventions Reducing Oxidative Stress on Behavioural and Gastrointestinal Problems in Autism Spectrum Disorder. Int. J. Dev. Neurosci. Off. J. Int. Soc. Dev. Neurosci..

[83] Hadders-Algra M. (2021). Early Diagnostics and Early Intervention in Neurodevelopmental Disorders-Age-Dependent Challenges and Opportunities. J. Clin. Med..

[84] Arand M., Mühlbauer R., Hengstler J., Jäger E., Fuchs J., Winkler L., Oesch F. (1996). A Multiplex Polymerase Chain Reaction Protocol for the Simultaneous Analysis of the Glutathione S-Transferase GSTM1 and GSTT1 Polymorphisms. Anal. Biochem..

[85] Tanito M., Kaidzu S., Takai Y., Ohira A. (2012). Status of Systemic Oxidative Stresses in Patients with Primary Open-Angle Glaucoma and Pseudoexfoliation Syndrome. PLoS ONE.

[86] Alagozlu H., Gorgul A., Bilgihan A., Tuncer C., Unal S. (2013). Increased Plasma Levels of Advanced Oxidation Protein Products (AOPP) as a Marker for Oxidative Stress in Patients with Active Ulcerative Colitis. Clin. Res. Hepatol. Gastroenterol..

[87] Collins A.R. (2004). The Comet Assay for DNA Damage and Repair: Principles, Applications, and Limitations. Mol. Biotechnol..

[88] Olive P.L., Banáth J.P. (2006). The Comet Assay: A Method to Measure DNA Damage in Individual Cells. Nat. Protoc..

[89] Gugliandolo A., Gangemi C., Calabrò C., Vecchio M., Di Mauro D., Renis M., Ientile R., Currò M., Caccamo D. (2016). Assessment of Glutathione Peroxidase-1 Polymorphisms, Oxidative Stress and DNA Damage in Sensitivity-Related Illnesses. Life Sci..

